# Artificial Neural Network Application in the Diagnosis of Disease Conditions with Liver Ultrasound Images

**DOI:** 10.1155/2014/708279

**Published:** 2014-09-16

**Authors:** Karthik Kalyan, Binal Jakhia, Ramachandra Dattatraya Lele, Mukund Joshi, Abhay Chowdhary

**Affiliations:** ^1^Systems Biomedicine Division, Haffkine Institute for Training Research and Testing, Parel, Mumbai, Maharashtra 400012, India; ^2^Research Advisory Council, Haffkine Institute for Training Research and Testing, Parel, Mumbai, Maharashtra 400012, India; ^3^Nuclear Medicine Department, Jaslok Hospital and Research Centre, Pedder Road, Mumbai, Maharashtra 400026, India; ^4^Ultrasound Department, Jaslok Hospital and Research Centre, Pedder Road, Mumbai, Maharashtra 400026, India

## Abstract

The preliminary study presented within this paper shows a comparative study of various texture features extracted from liver ultrasonic images by employing Multilayer Perceptron (MLP), a type of artificial neural network, to study the presence of disease conditions. An ultrasound (US) image shows echo-texture patterns, which defines the organ characteristics. Ultrasound images of liver disease conditions such as “fatty liver,” “cirrhosis,” and “hepatomegaly” produce distinctive echo patterns. However, various ultrasound imaging artifacts and speckle noise make these echo-texture patterns difficult to identify and often hard to distinguish visually. Here, based on the extracted features from the ultrasonic images, we employed an artificial neural network for the diagnosis of disease conditions in liver and finding of the best classifier that distinguishes between abnormal and normal conditions of the liver. Comparison of the overall performance of all the feature classifiers concluded that “mixed feature set” is the best feature set. It showed an excellent rate of accuracy for the training data set. The gray level run length matrix (GLRLM) feature shows better results when the network was tested against unknown data.

## 1. Introduction

**Figure 1:**
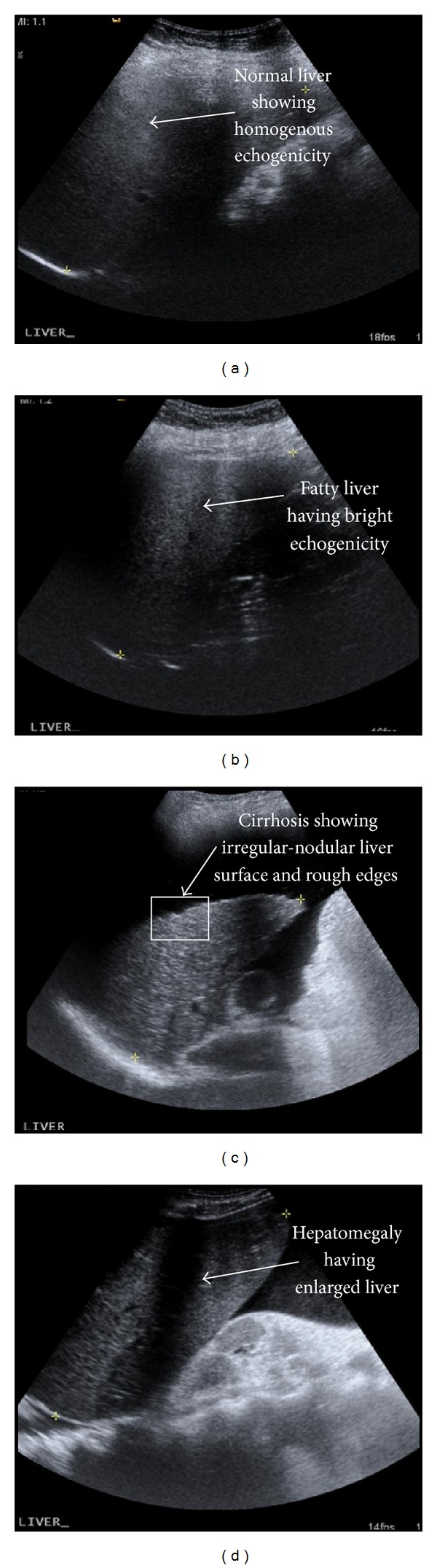
Ultrasound image of (a) normal liver, (b) fatty liver, (c) cirrhosis, and (d) hepatomegaly.

Ultrasound imaging modality is quite popular and most widely used modality for visualizing and studying the liver for any disease conditions without causing any pain or discomfort to the patient. Ultrasound liver imaging is widely used due to its noninvasive nature and low cost as compared to other imaging modalities. The diagnosis of various diseases is performed on the basis of various image features such as the echogenicity, legion shape, and echo texture. Liver imaging is one of the best techniques of early detection of liver diseases and early detection is very important because it saves patients from further ailments such as enlarged stomach filled with ascites fluid, bleeding varices, and encephalopathy or sometimes jaundice. Liver disease conditions such as fatty liver, cirrhosis, and hepatomegaly are known for producing distinctive echo patterns during US imaging as shown in Figure 1; however these images are also known to be visually challenging for interpreting them because of their imaging artifacts and speckle noise. As a result of it, the sonographers have to rely upon additional pathological tests [1–3].

A visual measure for diagnosing a liver is done by evaluating the liver echogenicity and the granular structure and surface echo-texture of liver. Texture analysis presents various image features, which characterize different liver conditions including normal and abnormal conditions. Texture analysis also provides some important information that cannot be obtained from visual examination of ultrasonic images. A texture analysis of each liver disease condition differs from other disease conditions as well as from the normal liver image. Normal liver ultrasound image is described as pyramidal with smooth surface and no lumps. The normal liver parenchyma is of homogeneous echogenicity [2]. Fatty liver ultrasound image shows liver parenchyma of hyperechogenicity. Fatty liver is reversible in its early stages; therefore an early detection is very essential [4, 5]. The ultrasound image of liver cirrhosis shows inhomogeneous echo texture and irregular-nodular liver surface [5]. Most gray levels of cirrhotic tissue appear darker than the normal tissue [6].

The granular structure of the tissue area can be examined to characterize it. This specific granular pattern of normal liver, cirrhotic liver, and fatty liver can be described as texture and thus “texture analysis” for tissue characterization may be used to study and correlate the physiological changes in the liver. Moreover this approach provides some important information that may not be obtained through visual interpretation of ultrasound images. These echo-patterns extracted from the images can be studied and processed for characterization of liver diseases [2].

Picture Archive and Communication Systems (PACS) are most widely used systems for medical image storage and retrieval. PACS are comprehensive networks of digital devices designed for digital image data management, image acquisition, data transmission, storage, image display, and management of diagnostic imaging studies, interfaces to printers and portable media, and communication routes to other electronic systems. PACS are usually based on DICOM (Digital Imaging and Communications in Medicine) standards and are comprehensive management systems for diagnostic imaging studies that are increasingly used in hospitals and other health care systems [7, 8].

An artificial neural network (ANN) is a nonlinear, computational, and mathematical model, comprised of densely interconnected simple processing elements called neurons. Artificial neural networks are inspired by information processing simulation in human brain by biological neurons. The main characteristics of neural networks are their ability to learn complex nonlinear input-output relationships, use sequential training procedures, and adapt themselves to the data. A key benefit of neural networks is that a model of the system can be built from the available data [9, 10].

The aim of this study was to investigate the image feature classifiers and to find the best feature classifier for the diagnosis of liver disease conditions using artificial neural network. The objectives included quantification of various features extracted from the ultrasound image and utilize them as an input to artificial neural networks towards the liver disease classification.

Previous studies conducted by Kadah et al. and Jeon et al. compared the analysis of statistical classifiers and neural network based classifiers generated using tissue characterization parameters from liver images [11, 12] by various feature extraction algorithms [11]. Another study conducted by Lee et al. established the classification of liver lesions such as cyst, hemangioma, and malignancies using the multiple regions of interests (ROIs) based feature selection methods. Here the liver lesion classification from the US images is heavily dependent upon certain characteristics (traits) such as internal echo, morphology, edge, echogenicity, and posterior echo enhancement. The proposed method seems to have achieved the enhanced and stable classification regardless of features used alongside outperforming the existing classification methods that are designed for focal liver lesions [13]. A study by Plesea-Condratovici et al. established the evaluating ability of a neural network based tool in the prediction of steatosis of liver, where the data has been collected from 100 patients and a data matrix is generated from that data and 10 variables are dedicated for the purpose. Out of the 10 variables 7 are input and 3 are output parameters. The results are validated against another study where the level of steatosis is known in the patients [14]. Study published by Gletsos et al. proposes the use of CAD system to classify the hepatic lesions such as hepatic cysts, hemangiomas, and hepatocellular carcinoma from CT images [15].

The rest of this paper is organized as follows.  Section 2 gives an outline of the overall methodology. Then, the processes of image acquisition, image preprocessing, and image processing (i.e., both feature extraction and feature selection processes) are explained under Sections 3, 4, and 5, respectively. We talk about the concept of artificial neural networks (in particular about validation, implementation, and testing aspects) under Section 6. We then talk about the results and discussion part under Section 7. Finally, the conclusion is given under Section 8.

## 2. Outline of Methodology

**Figure 2:**
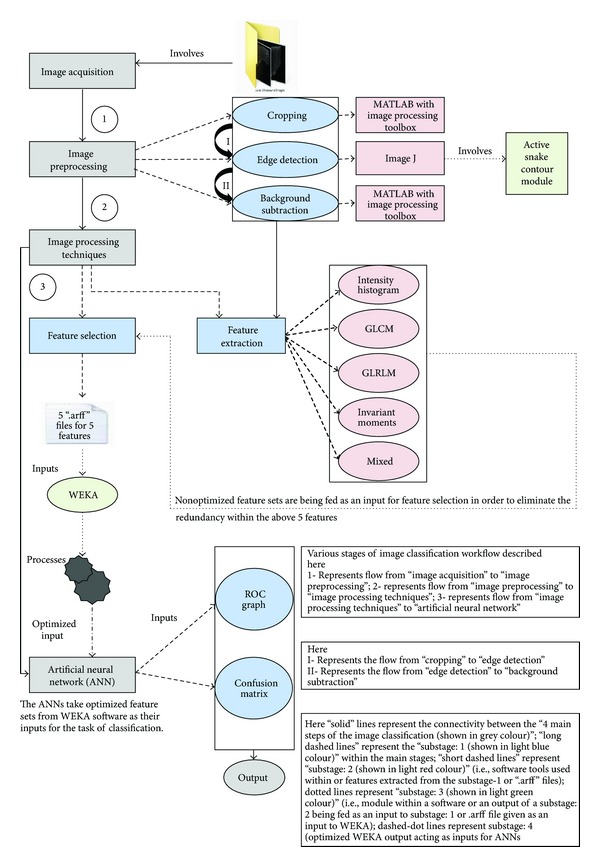
Image classification workflow utilising ANNs.

In this study, we acquired normal and diseased (i.e., abnormal) liver ultrasound images from “The Ultrasound Department at Jaslok Hospital and Research Centre.” Using MATLAB along with “image processing toolbox” these images are then subjected to three different image preprocessing techniques, namely, “cropping,” “edge detection,” and “background subtraction,” in order to accentuate the region of interest from the acquired images (i.e., liver). After image preprocessing, textural features such as “intensity histogram (IH)” [16, 17], “gray-level co-occurrence matrix (GLCM)” [18], “gray-level run length matrix (GLRLM)” [19], and “invariant moments (IM)” [20] were extracted from the preprocessed ultrasound images to calculate the adequate texture features. The features extracted from feature extraction phase were further processed (i.e., narrowed) utilizing “feature selection” method to obtain most significant and optimal features that represent the liver characteristics. WEKA software [21] was utilized within the feature selection phase to provide the selected significant features. These optimal features were then provided as an input to the neural network for classification. Artificial neural network employing a back-propagation algorithm [22] was utilized to classify the normal and abnormal liver disease conditions and to determine which feature classifier is best for classification. The performance of the neural network based classifier was determined using “confusion matrix” and “receiver operating characteristics (ROC)” curve analysis. These techniques as shown in Figure 2 are further elaborated in following subsections.

## 3. Image Acquisition

All B-mode ultrasound images were acquired from Jaslok Hospital and Research Centre, Mumbai. The patients' data report and their ultrasound images were initially analyzed and the images were shortlisted on the basis of the most prevalent disease condition amongst the acquired data, age of the patient, and severity of the disease. Here, the images of the patients containing ascites along with the liver disease conditions were eliminated from shortlisting as the selection parameters only focused on liver disease conditions. Overall, a total of 60 liver ultrasonic images were selected for this study. These included 30 cases of normal and 30 cases of abnormal liver sonograms. The 30 cases of abnormal liver are comprised of 10 cases of cirrhosis, 10 cases of fatty liver, and 10 cases of hepatomegaly. The images were acquired from both male and female subjects with a mean age group of 53 (±15) years. During image acquisition, the operator examined the whole liver area from different orientations and saved a single frame.

## 4. Image Preprocessing

**Figure 3:**
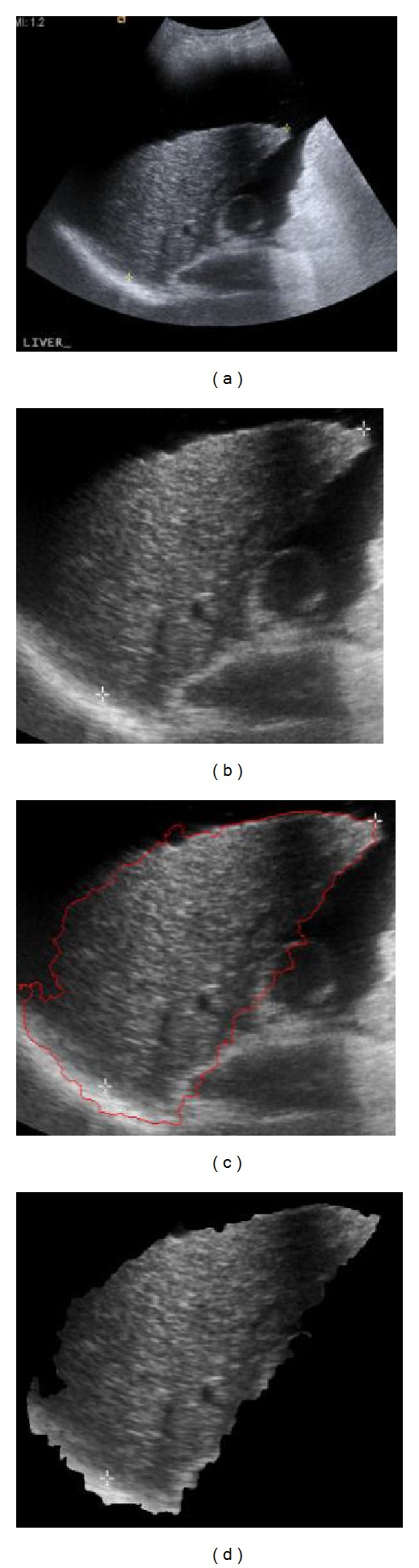
Workflow of image preprocessing step, (a) original ultrasound image, (b) image after cropping operation, (c) image after edge detection, and (d) image after background subtraction.

Image preprocessing techniques are used to select and enhance the region of interest and to eliminate erroneous data, which is of no interest from the acquired images. The images are subjected to three types of image preprocessing techniques such as cropping, edge detection, and background subtraction as shown in Figure 3.

### 4.1. Cropping

It is an operation, which is performed on acquired images to accentuate the region of interest (i.e., the liver) and to remove all the unwanted artifacts such as written labels and background noise from them. The cropping operation was done on all images by cutting out the rest of the area, which did not contain the liver, leaving a rectangular region consisting of only the region of interest. Cropping operation was performed on all images in MATLAB.

### 4.2. Edge Detection

It is one of the vital steps of image preprocessing. Although cropping operation extracts liver from the original image, it does not crop along the boundary of liver. Cropping is limited to a rectangular frame only whereas the shape as well as structure of liver is pyramidal; hence edge detection operation is necessary to define the edges of liver. Edge detection technique outlines the liver boundary in the image. In this work the segmentation is achieved by “Active Snake Contour” model, providing a contour over liver boundary. The active snake contour model is a semiautomatic model, where initializing a curve or a contour close to the boundary of the region of interest is carried out manually by the user and the model functions by deforming the contour through number of iterations to fit to the boundary of region of interest [23]. In this study, the edge detection was performed in “ImageJ” software using an “Absnake” plugin.

### 4.3. Background Subtraction

It is a method to eliminate unwanted intensity values which are outside the contour (edge) in the images (rectangular images with dimension of *m* × *n* that includes pyramidal liver) to avoid the calculation of these unwanted intensities that will be incorporated during extraction of feature parameters. Background subtraction is performed to avoid calculation of pixel intensities, which are outside the region of interest. Background subtraction was performed in MATLAB.

## 5. Image Processing

Texture is an image feature that provides important characteristics for surface and object identification from an image. Texture is characterized by the spatial distribution of gray levels in a neighborhood in an image. In texture analysis, the most difficult as well as important aspect is to define a set of meaningful features that explores the characteristics of the texture [24, 25]. Image processing techniques involve identifying these sets of essential features and extracting these features from the ultrasound image for further processing. “Feature extraction” and “feature selection” are two most vital steps of image processing and are explained under Sections 5.1 and 5.2 in detail.

### 5.1. Feature Extraction

Feature extraction is a critical step for ultrasonic liver classification. Feature extraction methodologies analyze the preprocessed images to extract the most prominent features that represent various sets of features based on their pixel intensity relationship and statistics. A set of four features (i.e., statistical texture features), namely, intensity histogram, gray-level co-occurrence matrix (GLCM), gray-level run-length matrix (GLRLM), and invariant moments, were extracted from each of the total 60 images in MATLAB using respective modules, which calculated parameters belonging to each set.

Each feature set comprises individual image parameters. Features derived from* intensity histogram* features include moments such as mean, standard deviation, average energy, entropy, skewness, and kurtosis [16, 17].* GLCM* features include autocorrelation, contrast, correlation, cluster prominence, cluster shade, dissimilarity energy, entropy, homogeneity (1), homogeneity (2), maximum probability, sum of squares, sum average, sum variance, sum entropy, difference variance, difference entropy, information measure of correlation (1), information measure of correlation (2), and inverse difference normalized [18]. The* GLRLM* features include short run emphasis (SRE), long run emphasis (LRE), run length nonuniformity (RLN), gray level nonuniformity (GLN), run percentage (RP), low gray-level run emphasis (LGRE), high gray-level run emphasis (HGRE), short run low gray-level emphasis (SRLGE), short run high gray-level emphasis (SRHGE), long run low gray-level emphasis (LRLGE), and long run high gray-level emphasis (LRHGE) [19]. The* Invariant moments* are *I*_1_, *I*_2_, *I*_3_, *I*_4_, *I*_5_, *I*_6_, and *I*_7_ and these moments are invariant under translation, changes in scale, and also rotation. So it describes the image despite its location, size, and rotation [20].

#### 5.1.1. Intensity Histogram Features

The intensity-level histogram is a function showing (for each intensity level) the number of pixels in the whole image, which have this intensity. Here *p* represents the “pixel intensity” and “*p*(*i*)” represents the pixel intensity of *I* value. The function *f*(*x*, *y*) can take discrete values *i* = 0,1,…, *N* − 1, where *N* is the total number of intensity levels in the image. We can calculate the individual features under this feature extraction technique utilizing the formulas as shown in Table 1.

#### 5.1.2. GLCM Features

Also termed as “spatial gray level dependency matrices,” it is one of the most widely used statistical tools for extracting texture information from images. The GLCM of a 2D (i.e., *N*_*x*_ × *N*_*y*_) image containing pixels with gray levels (0,1,…, *G* − 1) is also a 2D matrix “*P*(*i*, *j*)”, where each and every matrix element depicts the probability of joint occurrence of intensity levels “*k*” and “1” at a certain distance “*d*” and an angle “*θ*”. Here “*p*(*i*, *j*)” is the (*i*, *j*)th entry in a normalized GLCM. The mean (*μ*_*x*_, *μ*_*y*_) and standard deviation (*σ*_*x*_, *σ*_*y*_) for the rows and columns of the 2D matrix can be calculated using the formulas that are shown in Table 2. We can also calculate the various individual features under this feature extraction technique utilizing the formulas provided in Table 2.

#### 5.1.3. GLRLM Features

Grey-level run-length matrix (GLRLM) is a matrix from which the texture features can be extracted for texture analysis. For a given 2D image, GLRLM is a 2D matrix in which element “*p*(*i*, *j*)” gives the total number of consecutive runs of length “*j*” at grey level “*i*”. Here “*M*” represents the number of gray levels and “*N*” represents the maximum run length (here a run length is considered to be a number of neighboring pixels that possess the same grey intensity in a particular direction). From this matrix almost 11 scalar parameters can be computed which analyze the image texture [26] and these parameters are provided in Table 3.

#### 5.1.4. Invariant Moments Features

The idea of utilizing moments within shape recognition became popular in 1962 when Hu utilised algebraic invariants to derive a set of invariants. Hu's 7 moment invariants are invariant under translation, changes in scale, and also rotation. So it describes the image despite its location, size, and rotation. The moment invariants are generally specified in terms of normalized central moments. Here, the central moments are depicted by *μ*_*pq*_, the raw moments are defined by *μ*_*ii*_ [27], and these invariants are shown in Table 4.

#### 5.1.5. Mixed Features

Mixed features are a combined set of all features, that is, intensity histogram features, GLCM features, GLRLM features, and invariant moment features. Mixed features are a combination of these features including a total of 47 features including all 46 features of intensity histogram features, GLCM features, GLRLM features, and invariant moment features and length of the liver. Mixed features set is created to check significance of all attributes within all features and to serve as an input to neural network.

### 5.2. Feature Selection

While feature extraction techniques are applied to extract as many image parameters as possible that identify liver characteristics, a feature selection algorithm is necessary to select few of those extracted features which are most significant and which describe the liver characteristics the best.

A total of 46 features were extracted in feature extraction process from each image but all of these features cannot be supplied to the neural network because the number of features is high. Although each feature is important in classification only few of these features are very significant in classifying and identification of the disease conditions. Therefore instead of using all of these features as input, only those features, which have high significance, were selected.

There are many feature selection algorithms used in this process and each performed search in their unique way. Most of the feature selection algorithms involve a search method throughout the whole space. Many search methods calculate individual feature's significance and rank them accordingly. These methods also provide best features from a given set. Waikato Environment for Knowledge Analysis (WEKA) software gives a variety of feature selection options that include “heuristic,” “genetic,” and “Bayesian” algorithms. Here, feature selection was performed using WEKA software version 3.6.9 [21].

WEKA is compatible with and recognizes only Attribute-Relation file format (i.e., “.arff file format”); therefore “.arff file” was generated containing feature information of all images (normal as well as abnormal). Along with “.arff file” consisting of four features, one more “.arff file” called mixed features was created containing all the 46 features and the length of liver parameter. Feature selection process incorporated methods like genetic search method, random search, rank search, and so forth to select the best attributes amongst a large set of features. These methods select features on the basis of their ability to correctly identify the pattern in training. WEKA provides option of varied search methods for selection of attributes, out of which we used only two search methods, namely, “RandomSearch” which performs a random search in the space of attribute subsets and “GeneticSearch” which performs a search using the simple genetic algorithm. The feature selection algorithms such as “RandomSearch” and “GeneticSearch” of WEKA software were used to generate the results as shown in Table 5. These optimal features were selected by WEKA software for serving as an input in neural network.

## 6. Artificial Neural Network

An artificial neural network builds a model of existing system and learns from the previous or known samples and trains the network to achieve target with minimum error [22].

### 6.1. Implementation, Validation, and Testing

**Figure 4:**
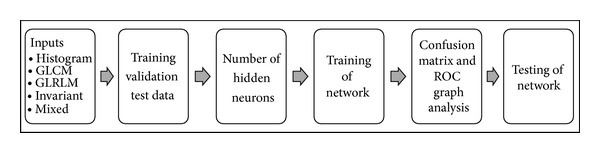
Workflow of implementation of artificial neural network.

The features extracted from the images after the feature selection process act as an input to the neural network as shown in Figure 4. To perform the analysis for image classification, the back propagation algorithm has been shortlisted and is implemented using MATLAB's Neural Network Pattern Recognition Tool (nprtool). The back propagation algorithm was chosen due to the networks ability to learn and store immense amounts of mapping relations of input-output model without the need for prior disclosure of mathematical equations pertaining to these mapping relations. The algorithm also regulates the network's weight and threshold values in order to obtain minimum error sum of square [28]. The designed neural network classifier used a two-layer feed-forward back propagation network. Two-layer feed-forward network can be best defined as a network with sigmoid hidden and output neurons. The network was trained with scaled conjugate gradient back propagation [22, 29].

To train the network, the input data and target data need to be fed into the network. The network then divides the input sample data into three different samples, which are training, validation, and testing samples. The training samples are used to train the network, and the network is adjusted according to its error. The validation samples are used to measure network generalization and to halt the training when generalization stops improving. Testing samples are then used to provide an independent measure of the network performance during and after training. If the error of the network is still large, the network can be retrained back as to get more accurate and efficient result [22, 29]. From the training dataset, we utilized 80% of data for training, 10% of data for validation, and 10% of data for testing purposes, while we utilized 20 samples of normal condition and 20 samples of abnormal condition to create the testing dataset to test the efficiency of the artificial neural network.

Confusion matrix as well as ROC graph depicts the overall classification rate and accuracy of the network. If the overall classification rate and the accuracy are high, it signifies that the network was successful in correctly classifying the two classes. After training the network for sufficient number of epochs till the network is perfectly trained having low MSE and less misclassifications, confusion matrix and ROC graph were plotted to measure the true positive rate, that is, sensitivity, true negative rate, that is, specificity, false positive rate, false negative rate, and accuracy of the network [29].

Analysis of ROC graph and confusion matrix of the trained network are generally more than enough for evaluating the designed neural network classifier's accuracy. There is an additional option to test network on more data and then decide the quality of the network's performance. Additional tests were performed on test input dataset, comprised of a smaller sample set to evaluate the network's performance on test data. “MSE” and “percent error” provided the mean squared error and difference between the output and target test data, respectively.

## 7. Results and Discussion

The performance of the designed neural network classifier is measured in terms of accuracy. This term refers to the ability of the model to correctly predict the class of new unseen data. Classification accuracy is calculated by determining the percentage of cases in which the test sets are correctly classified. The performance of the neural network was calculated by analysis of confusion matrix and the receiver operator characteristic curve (ROC).

*Confusion Matrix.* The diagonal cells show the number of classes that were correctly classified and the off diagonal cells show the misclassified cases. The blue cell in the bottom right shows the total percent of correctly classified cases (in green) and the total percent of misclassified cases (in red) [29].

*ROC Graph.* The colored lines in this graph represent the ROC curves for each of the two output categories. The ROC curve is a plot of the true positive rate (sensitivity) versus the false positive rate (1-specificity) as the threshold is varied. A perfect test would show points in the upper-left corner, with 100% sensitivity and 100% specificity [29].

**Figure 5:**
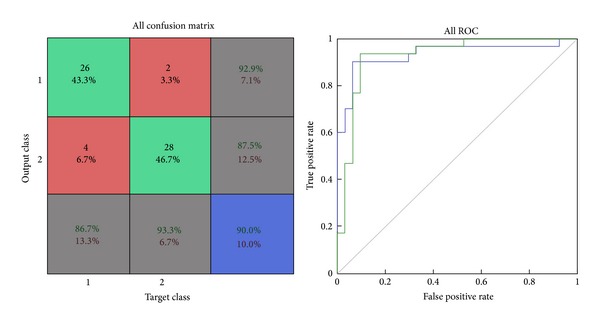
Confusion matrix and ROC plot of GLRLM training data.

Results of the training data show that selected GLRLM features yield an accuracy of 90%, sensitivity of 86.7%, specificity of 93.3%, false positive rate computed of 6.7%, false negative rate computed of 13.3%, and misclassification rate of 10%. Size of the input dataset loaded in the network was of 60 samples. Of total 60 samples, 54 samples were correctly classified and 6 samples were misclassified by this network as shown in Figure 5. ROC graph shows the plotting of true positive rate against false positive rate (1-specificity). ROC graph of this network shows an excellent classification between the two categories as the curves lie between the diagonal and the upper-left corner but mainly towards the upper-left corner as shown in Figure 5.

**Figure 6:**
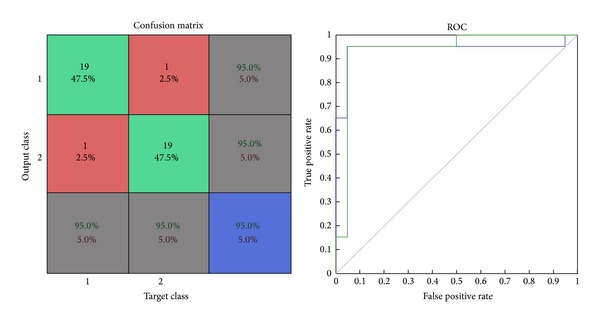
Confusion matrix and ROC plot of GLRLM testing data.

The results of the testing data show that GLRLM histogram features yield an accuracy of 95%, sensitivity of 95%, specificity of 95%, false positive rate computed of 5%, false negative rate computed of 5%, and misclassification rate of 5%. Size of the input dataset loaded in the network was of 40 samples. Out of the total 40 samples, 38 samples were correctly classified and 2 samples were misclassified by this network as shown in Figure 6. ROC graph shows the plotting of true positive rate against false positive rate (1-specificity). ROC graph of this network shows a perfect classification between the two categories as the curves lie in the region of upper-left corner and they are very far from the diagonal as shown in Figure 6.

**Figure 7:**
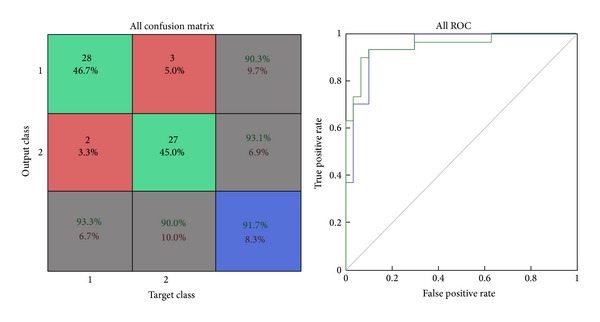
Confusion matrix and ROC plot of mixed features training data.

The results of the training data show that selected mixed features yield an accuracy of 91.67%, sensitivity of 93.33%, specificity of 90%, false positive rate computed of 10%, false negative rate computed of 6.7%, and misclassification rate of 8.33%. Size of the input dataset loaded in the network was of 60 samples. Out of the total 60 samples, 55 samples were correctly classified and 5 samples were misclassified by this network as shown in Figure 7. ROC graph shows the plotting of true positive rate against false positive rate (1-specificity). ROC graph of this network shows a perfect and effective classification between the two categories as the curves lie towards the upper-left corner as shown in Figure 7.

**Figure 8:**
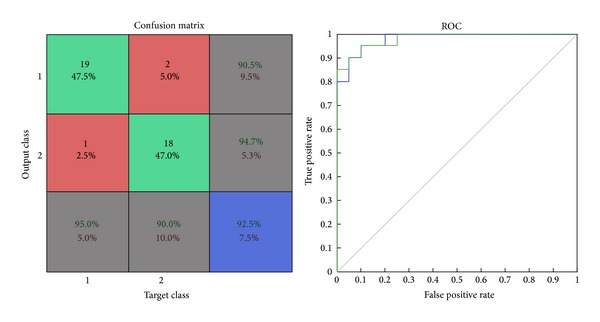
Confusion matrix and ROC plot of mixed features testing data.

The results of the testing data show that mixed features yield an accuracy of 92.5%, sensitivity of 95%, specificity of 90%, false positive rate computed of 10%, false negative rate computed of 5%, and misclassification rate of 7.5%. Size of the input dataset loaded in the network was of 40 samples. Out of the total 40 samples, 37 samples were correctly classified and 3 samples were misclassified by this network as shown in Figure 8. ROC graph shows the plotting of true positive rate against false positive rate (1-specificity). ROC graph of this network shows a perfect classification between the two categories, as the curves lie in the region of upper-left as shown in Figure 8.

The results showed that the selected mixed features yielded an accuracy of around 91.67% on the training set as compared to GLRLM features and GLCM features, which yielded an accuracy of around 90% and 86.7%, respectively, on the training set. The histogram feature gives around 77.5% and the invariant moments give around 65% of accuracy on training set as shown in Table 6.

**Figure 9:**
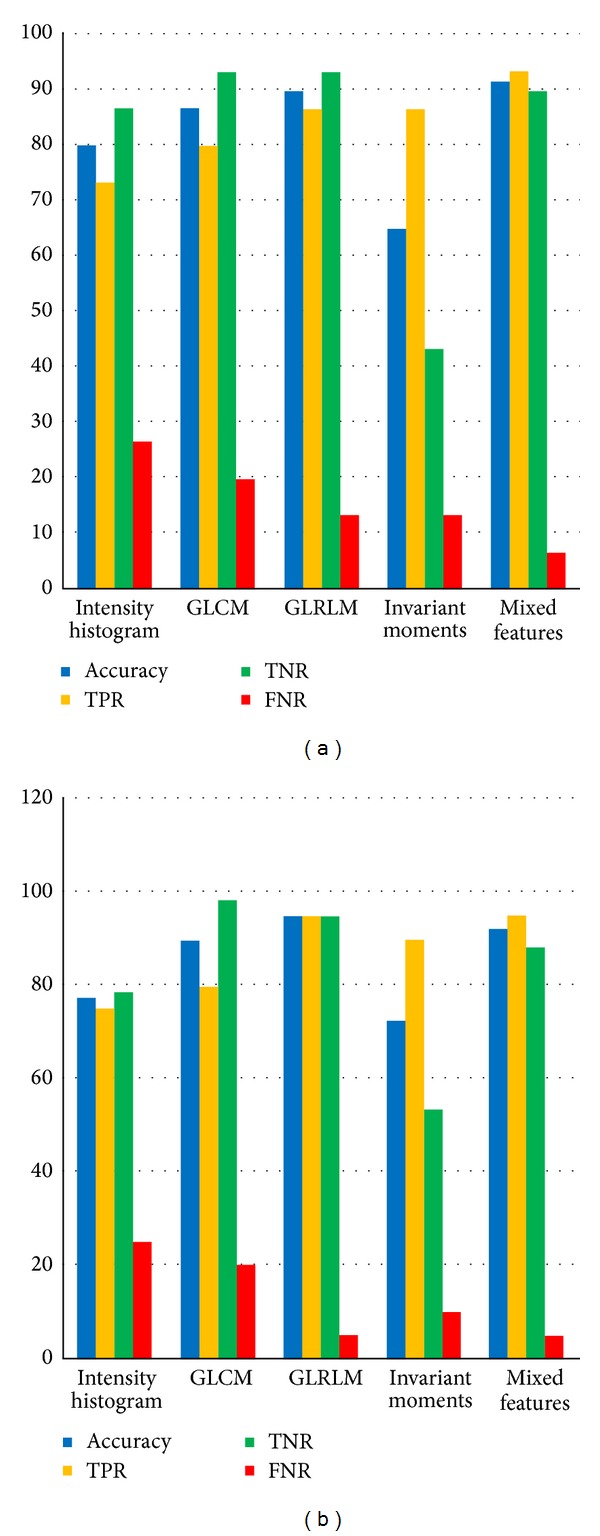
(a) Overall performance analysis of training dataset and (b) overall performance analysis of testing dataset.

These results indicate that the highest accuracy is achieved by the “mixed features set” with the accuracy being 91.7% by the “training dataset” and 92.5% by the “testing dataset” as shown in Figure 9. Hence the selected features of the mixed feature set such as homogeneity (homom), sum of averages (savgh), difference variance (dvarh), information measure of correlation-1 (inf1h), information measure of correlation-2 (inf2h), inverse difference normalized (indnc), short run emphasis (SRE), short run high gray-level emphasis (SRHGE), and length (l) show excellent accuracy.

Along with the mixed features, the gray-level run length matrix features also showed an excellent accuracy. Training set yielded an accuracy of 90%, but its testing dataset showed a better accuracy of 95%, that is, greater than both training and testing data accuracy of the mixed features. For testing the network against test data, GLRLM features gave better result. A low false negative rate decreases the possibility of misclassification. Both mixed and GLRLM features yielded a low false negative rate as shown in Table 6. The false positive rate of both mixed and GLRLM features is 5% and it is lower than the other networks.

## 8. Conclusion

In this study, five feature classifiers have been investigated for diagnosing the liver disease conditions. The accuracy of the classifier was based upon the feature set used, selected training samples, and the classifier's ability to learn from the training samples. From the above results, we have achieved our objective in finding the best classifier for liver disease diagnosis. Five sets of features such as GLCM, intensity histogram, GLRLM, invariant moments, and mixed features were extracted. These features were then selected and trained in neural network to determine the best set of features, which can determine the presence of disease conditions in the liver. A comparative approach revealed that both GLRLM and mixed feature set showed excellent accuracy in training as well as testing.

## Figures and Tables

**Table 1:** Features corresponding to intensity histogram.

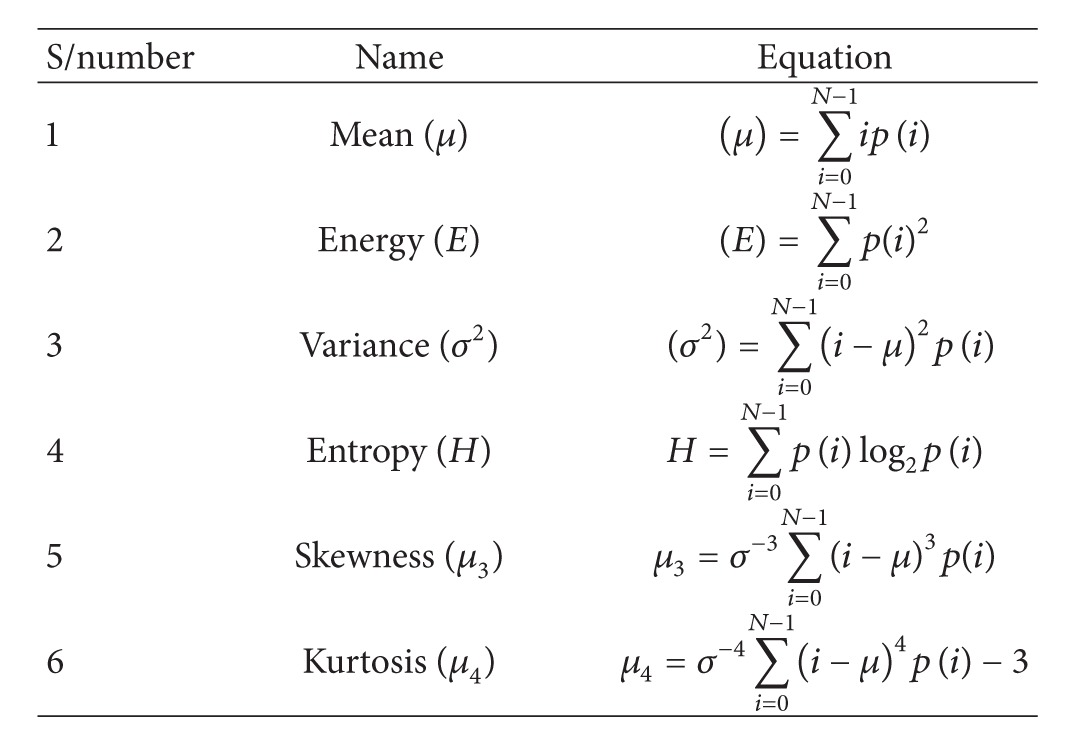

**Table 2:** Features of GLCM.

S/number	Name	Equation
1	Mean (*µ*_*x*_, *µ*_*y*_)	${µ}_{x}=\underset{i}{\sum }\underset{j}{\sum }i\cdot p(i,j)$; ${µ}_{y}=\underset{i}{\sum }\underset{j}{\sum }j\cdot p(i,j)$

2	Standard deviations (*σ*_*x*_, *σ*_*y*_)	$\sigma_{x}=\underset{i}{\sum }\underset{j}{\sum }{\left(i-\mu_{x}\right)}^{2}\cdot p\left(i,j\right)$; $\sigma_{y}=\underset{i}{\sum }\underset{j}{\sum }{\left(i-\mu_{y}\right)}^{2}\cdot p\left(i,j\right)$

2.1	Autocorrelation (*f*_1_)	$f_{1}=\underset{i}{\sum }\underset{j}{\sum }\left(ij\right)\cdot p\left(i,j\right)$

2.2	Contrast (*f*_2_)	$f_{2}=\overset{N_{9}-1}{\underset{n=0}{\sum }}n^{2}\left\{\overset{N_{g}}{\underset{i=1}{\sum }}\overset{N_{G}}{\underset{j=1}{\sum }}p\left(i,j\right)|\left|i-j\right|=n\right\}$

2.3	Correlation (*f*_3_)	$f_{3}=\frac{\left[\sum_{i}\sum_{j}\left(ij\right)p\left(i,j\right)-{µ}_{x}{µ}_{y}\right]}{\left[\sigma_{x}\sigma_{y}\right]}$

2.4	Cluster prominence (*f*_4_)	$f_{4}=\underset{i}{\sum }\underset{j}{\sum }{\left(i+j-\mu_{x}-\mu_{y}\right)}^{4}p\left(i,j\right)$

2.5	Cluster shade (*f*_5_)	$f_{5}=\underset{i}{\sum }\underset{j}{\sum }{\left(i+j-\mu_{x}-\mu_{y}\right)}^{3}p(i,j)$

2.6	Dissimilarity (*f*_6_)	$f_{6}=\underset{i}{\sum }\underset{j}{\sum }\left|i-j\right|\cdot p(i,j)$

2.7	Energy (*f*_7_)	$f_{7}=\underset{i}{\sum }\underset{j}{\sum }p{\left(i,j\right)}^{2}$

2.8	Entropy (*f*_8_)	$f_{8}=-\underset{i}{\sum }\underset{j}{\sum }p(i,j)\log\left(p\left(i,j\right)\right)$

2.9	Homogenecity (*f*_9_)	$f_{9}=\underset{i}{\sum }\underset{j}{\sum }\frac{1}{1+{\left(i+j\right)}^{2}}p(i,j)$

2.10	Maximum probability (*f*_10_)	$f_{10}=\underset{i,j}{\max}p(i,j)$

2.11	Sum of squares (*f*_11_)	$f_{11}=\underset{i}{\sum }\underset{j}{\sum }{\left(i-\mu \right)}^{2}p(i,j)$

2.12	Sum average (*f*_12_)	$f_{12}=\overset{2N_{g}}{\underset{i=2}{\sum }}ip_{x+y}(i)$

2.13	Sum variances (*f*_13_)	$f_{13}=\overset{2N_{g}}{\underset{i=2}{\sum }}{\left(i-f_{14}\right)}^{2}ip_{x+y}(i)$

2.14	Sum entropy (*f*_14_)	$f_{14}=\overset{2N_{g}}{\underset{i=2}{\sum }}p_{x+y}(i)\log\left\{p_{x+y}\left(i\right)\right\}$

2.15	Difference variance (*f*_15_)	*f*_15_ = Variance of *p*_*x*−*y*_

2.16	Difference entropy (*f*_16_)	$f_{16}=-\overset{N_{9}-1}{\underset{n=0}{\sum }}p_{x-y}\left(i\right)\log\left\{p_{x-y}(i)\right\}$

2.17 2.18	Information measures of correlation-1 (*f*_17_) Information measures of correlation-2 (*f*_18_)	$f_{17}=\frac{HXY-HXY1}{\max\left\{HX,HY\right\}}$ *f*_18_ = (1 − exp⁡[ − 2.0(*HXY*2 − *HXY*)])^1/2^ *where HX and HY are entropies of P*_*X*_ *and P*_*Y*_ * * $HXY=\underset{I}{\sum }\underset{J}{\sum }p\left(i,j\right)\log\left(p\left(i,j\right)\right)$ $HXY1=\underset{I}{\sum }\underset{J}{\sum }p\left(i,j\right)\log\left\{p_{x}\left(i\right)p_{y}\left(j\right)\right\}$ $HXY2=\underset{I}{\sum }\underset{J}{\sum }p_{x}\left(i\right)p_{y}\left(j\right)\log\left\{p_{x}\left(i\right)p_{y}\left(j\right)\right\}$

2.19	Inverse difference (*f*_19_)	Same as homogenecity

2.20	Inverse difference normalized [INN] (*f*_20_)	*C*_*ij*_ * = the co-occurrence probability between grey levels i and j is defined as* $C_{ij}=\frac{P_{ij}}{\sum_{i,j=1}^{G}p_{ij}}$ $f_{20}=\overset{G}{\underset{i,j=1}{\sum }}P_{IJ}\frac{C_{ij}}{1+{\left|i-j\right|}^{2}/G^{2}}$

2.21	Inverse difference moment normalized (*f*_21_)	$f_{21}=\overset{G}{\underset{i,j=1}{\sum }}P_{IJ}\frac{C_{ij}}{1+{\left(i-j\right)}^{2}/G^{2}}$

**Table 3:** Features of GLRLM.

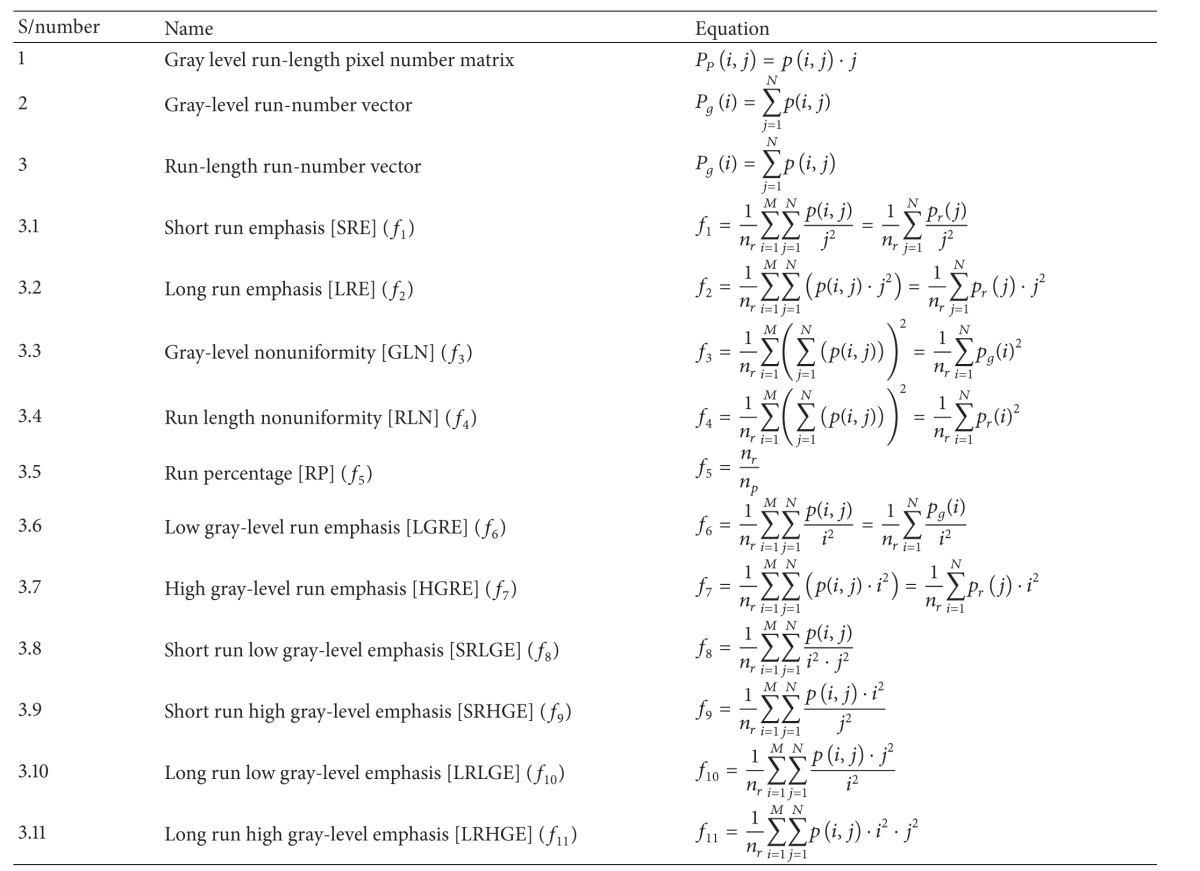

**Table 4:** Features corresponding to invariant moments.

S/number	Name	Equation
1	Raw moments (*M*_*ij*_)	$M_{ij}=\underset{x}{\sum }\underset{y}{\sum }x^{i}y^{j}p(x,y)$

2	Central moments (*μ*_*pq*_)	*f*(*x*, *y*) is a digital image; *p*, *q* are raw moments; $\overset{-}{X}=M_{10}/M_{00}$ and $\overset{-}{Y}=M_{01}/M_{00}$ are the components of the centroid: $\mu_{pq}=\overset{p}{\underset{m}{\sum }}\overset{q}{\underset{m}{\sum }}\left(\begin{array}{c} p\\ m \end{array}\right)\left(\begin{array}{c} q\\ n \end{array}\right){\left(-\overset{-}{x}\right)}^{p-m}{\left(-\overset{-}{y}\right)}^{q-n}M_{mn}$

3	Invariant moments	$\eta_{ij}=\frac{\mu_{ij}}{\mu_{00}^{\left(1+(i+j)/2\right)}}$ *I*_1_ = *η*_20_ + *η*_02_, *I*_2_ = (*η*_20_−*η*_02_)^2^ + 4*η*_11_^2^, *I*_3_ = (*η*_30_−3*η*_12_)^2^ + (3*η*_21_−3*η*_03_)^2^, *I*_4_ = (*η*_30_+*η*_12_)^2^ + (*η*_21_−*η*_03_)^2^, *I*_5_ = (*η*_30_ − 3*η*_12_)(*η*_30_ + *η*_12_)[(*η*_30_+3*η*_12_)^2^ − (3*η*_21_+3*η*_03_)^2^] + (3*η*_21_ − *η*_03_)(*η*_21_ + *η*_03_)[3(*η*_30_+*η*_12_)^2^ − (*η*_21_+*η*_03_)^2^] *I*_6_ = (*η*_20_ − *η*_02_)[(*η*_30_+*η*_12_)^2^ − (*η*_21_+*η*_03_)^2^] + 4*η*_11_(*η*_30_ + *η*_12_)(*η*_21_ + *η*_03_), *I*_7_ = (3*η*_21_ − 3*η*_03_)(*η*_30_ + *η*_12_)[(*η*_30_+*η*_12_)^2^ − (3*η*_21_+3*η*_03_)^2^] − (*η*_30_ − 3*η*_21_)(*η*_21_ + *η*_03_)[3(*η*_30_+*η*_12_)^2^ − (*η*_21_+*η*_03_)^2^]

**Table 5:** Features selected by WEKA software.

S/number	Feature category	Number of features before feature selection	Number of features after feature selection	Selected features
1	Intensity histogram	6	4/6	Variance, Skewness, Kurtosis, and Entropy

2	GLCM	22	11/22	Contrast, correlation-1, correlation-2, cluster shade, homogeneity, maximum probability, sum of squares: variance, sum variance, difference entropy, information measure of correlation-1, and information measure of correlation-2

3	GLRLM	11	6/11	Short run emphasis, gray-level nonuniformity, low gray-level run emphasis, high gray-level run emphasis, short run high gray-level emphasis, and long run high gray-level emphasis

4	Invariant moments	7	4/7	*I*_2_, *I*_3_, *I*_4_, and *I*_6_

5	Mixed features	47	9/47	Homogeneity, sum of average, difference variance, information measure of correlation-1, information measure of correlation-2, inverse difference normalized, short run emphasis, short run high gray-level emphasis, and length

**Table 6:** Performance analysis of all features.

FEATURE	Training (%)				Testing (%)			
	Accuracy	TPR	TNR	FNR	Accuracy	TPR	TNR	FNR
Intensity histogram	80	73.3	86.7	26.7	77.5	75	80	25
GLCM	86.7	80	93.3	20	90	80	100	20
GLRLM	90	86.7	93	13.3	95	95	95	5
Invariant moments	65	86.7	43.3	13.3	72.5	90	55	10
Mixed feature	91.7	93.3	90	6.7	92.5	95	90	5
